# Tractographic analysis of proprioceptive and pain pathways in individuals with degenerative scoliosis

**DOI:** 10.1097/MD.0000000000049717

**Published:** 2026-07-10

**Authors:** Yasin Göktürk, Şule Göktürk, Sabri Batin, Tahir Fatih Dikici, Ahmet Payas

**Affiliations:** a Department of Neurosurgery, Kayseri City Education and Training Hospital, Kayseri, Turkey; b Department of Orthopedics and Traumatology, Kayseri City Education and Training Hospital, Kayseri, Turkey; c Department of Therapy and Rehabilitation, Vocational School of Health Services, Alanya Alaaddin Keykubat University, Antalya, Turkey; d Department of Anatomy, Amasya University, Faculty of Medicine, Amasya, Turkey.

**Keywords:** brain, degenerative scoliosis, formatio reticularis, magnetic resonance imaging, pain, tractography

## Abstract

Although increased pain and balance problems in individuals with degenerative scoliosis (DS) are well documented, the associated changes in the central nervous system remain unclear. This study aimed to investigate the impact of postural imbalance and pain in individuals with DS on the central sensory pathways responsible for proprioception and pain modulation. In this context, tractography results of the proprioceptive and pain-modulating pathways were compared between individuals with DS and healthy controls. A total of 38 individuals with DS and 38 healthy individuals were included in the study. Bilateral tractography analyses of relevant pathways were conducted using brain magnetic resonance imaging data and DSI Studio software. Statistical analyses were performed using IBM SPSS 23.0, with *P* values <.05 considered statistically significant. No significant differences were found between groups in terms of age, weight, height, sex, or BMI (*P* > .05). Whole-brain average fiber length, mean diffusivity, axial diffusivity, and radial diffusivity values were significantly higher in the DS group compared to the control group (*P* < .05). Tractography metrics of the lemniscus medialis were significantly lower in individuals with DS (*P* < .05). In the left reticular formation, average fiber length, fiber volume, fiber area, and fiber proportion values were significantly higher in the DS group (*P* < .05). In addition, visual analog scale scores for pain were significantly higher in the DS group compared to controls (*P* < .05). This study demonstrates that increased pain perception and postural imbalance in individuals with DS may be associated with alterations in central sensory pathways, particularly the lemniscus medialis and reticular formation.

## 1. Introduction

Degenerative scoliosis (DS) is a spinal deformity characterized by degenerative changes exceeding 10° in the spine, which usually occurs after the age of 50.^[1,2]^ DS develops simultaneously with disc degeneration, loss of stability, and asymmetric collapse of the disc and is mostly seen in the lumbar region. Patients with DS present with back pain, lower extremity pain, and functional impairments due to progressive deformity.^[1]^

It has been shown that back pain is more common, severe, and prolonged in patients with scoliosis compared to individuals without scoliosis.^[3]^ In patients with DS, the pain is usually asymmetrical and frequently seen in the areas where the deformity is most pronounced.^[4]^ The main sources of pain in lumbar degenerative scoliosis are internal disc instability, facet joint degenerative arthritis, and foraminal stenosis.^[5,6]^

Various anatomical structures play a role in the regulation of pain perception in the central nervous system. The reticular formation (RF) plays a critical function in the coordination and modulation of nociception.^[7]^ It is also known that patients with DS have altered postural balance mechanisms.^[8]^ It has been observed that in patients with spinal deformities, various postural adaptations develop in the spine, pelvis, and lower extremities to compensate for the anterior shift in the gravity line.^[9]^ In individuals with DS, step length decreases, and the range of motion of the extremities and trunk rotation become asymmetrical during walking.^[10]^

Balance is a complex process achieved through the integration of visual, vestibular, and somatosensory systems.^[11]^ A disorder occurring in any of these systems negatively affects postural control.^[12]^ The brain maintains spinal alignment and balance based on proprioceptive signals from different body parts.^[13]^ Impaired proprioception may negatively impact movement accuracy and body positioning during functional tasks.^[14]^ Proprioceptive senses are perceived through mechanoreceptors in the muscles, tendons, joint capsules, and ligaments, and these signals are transmitted through the spinal pathways of the central nervous system.^[12]^

Advanced imaging techniques provide important contributions to the study of structural and functional changes in the central nervous system. Magnetic resonance imaging (MRI) allows detailed examination of anatomical structures. Diffusion tensor imaging provides information on the myelin structure and axonal integrity of white matter tracts.^[15]^

This study aims to investigate the effects of pain and postural balance problems seen in individuals with degenerative scoliosis on the pain and proprioceptive sensory pathways in the central nervous system. In this context, tractography results of the pathways that play a role in pain modulation and transmit proprioceptive sensation will be compared between individuals with degenerative scoliosis and healthy individuals.

## 2. Materials and methods

### 2.1. Study design

The study was conducted at a single center as a cross-sectional cohort. Ethical approval for the research was granted by the Local Ethics Committee of Nuh Naci Yazgan University, Kayseri, Türkiye (decision number 2023-009/005, dated 23/10/2023). The study was conducted in accordance with the Declaration of Helsinki, and written consent was obtained from each participant before inclusion in the study. The sample of this study was determined as α = 0.05, β = 0.19, effect size = 1.08 by posthoc power analysis using G power.

The DS group consisted of 38 patients, while the control group included 38 healthy individuals. Inclusion criteria for the DS group were individuals over 50 years of age with clinical and radiological signs of spinal degeneration (e.g., osteophytes and Schmorl nodes) and a Cobb angle exceeding 20°. Although these patients were identified as potential surgical candidates due to the severity of their spinal deformity and persistent symptoms, they were followed conservatively in our outpatient clinic between January 2024 and January 2025. To eliminate the potential confounding effects of surgical interventions on central nervous system (CNS) pathways and diffusion tensor imaging metrics, individuals with a history of prior spinal surgery or deformity correction procedures were excluded. Furthermore, patients receiving pharmacological treatment or any other active therapy for pain management at the time of the study were excluded to ensure a baseline assessment of neural pathways.

The following criteria were used to exclude participants from all groups: the presence of any mental health problem, neurological, psychiatric, muscular, or rheumatic disease; a history of head injury; a history of headache; a history of back injury; weakness or numbness in any of the extremities; and the presence of space-occupying lesions in the brain. Brain MRI results were also used to determine eligibility.

Visual analogue scale (VAS) was used to measure the intensity or frequency of pain in the participants. On the scale, pain intensity is expressed by numbers between 0 and 10 on the line segment, where 0 indicates no pain and 10 indicates severe pain. Before the study, the VAS was explained to all participants in detail, and they were asked to mark according to the level of pain they felt.

### 2.2. Obtaining brain diffusion magnetic resonance images

Brain MRI procedures of the volunteers participating in the study were performed with a 3T (Tesla) Siemens Magnetom Skyra (Netherlands) MRI device.

- T1 weighted MPRAGE sequence: Sagittal, repetition time (TR) = 2300 ms, echo time (TE) = 3.4 ms, field of view = 250 mm, matrix: 256 × 256, slice thickness = 1 mm.
- DTG: Axial, TR = 4900 ms, TE = 95 ms, number of slices=36, field of view = 230 mm, matrix: 128 × 128, slice thickness = 3.5 mm, averages = 3, *b* = 0.1000 s/mm^2^, 20 diffusion directions. The resulting images were converted to DICOM format and saved.

### 2.3. Data processing

**Figure 1.**
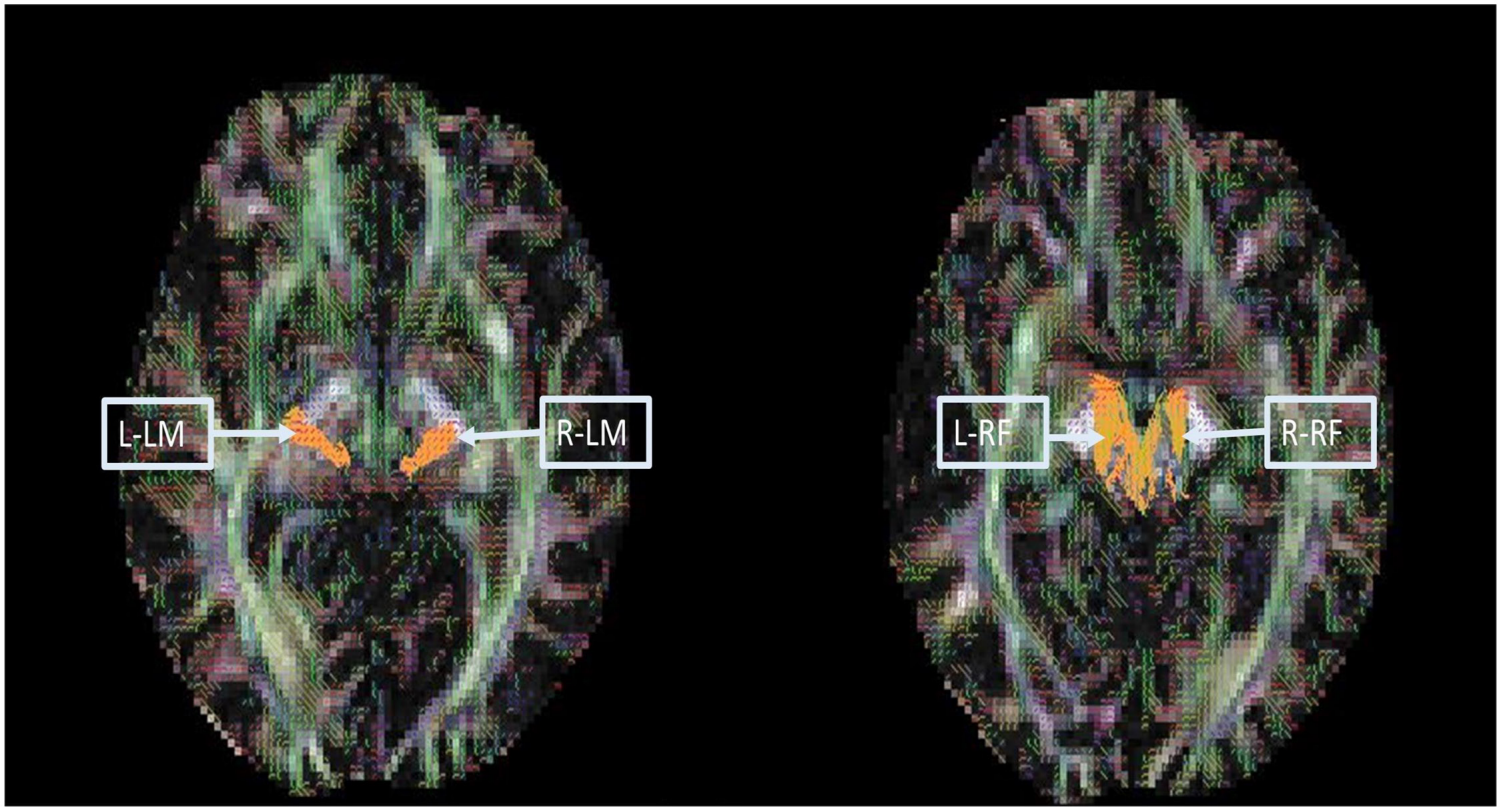
Display of all fibers in the pain path with the DSI Studio program. L-LM = left lemniscus medialis, L-RF = left reticular formation, R-LM = right lemniscus medialis, R-RF = right reticular formation.

A study on lemniscus medialis (LM) and RF tractography was performed using DSI Studio software. The tractography process was initiated for each participant by establishing the “Fiber Tracking” settings (Fig. 1). The threshold was set to 0.20, the angular threshold to 70°, the smoothing value to 0.50, the shortest tract length to 10 mm, the longest tract length to 1000 mm, and the termination criterion to 100,000 fibers. In order to reduce the margin of error associated with the tractography process, the “Tractography Atlas Tool,” which is included in the DSI Studio software, was employed.

As a consequence of the aforementioned procedure, the following data were obtained for each participant: right and left LM and RF volume, volume ratio, superficial area, superficial area ratio, total number of fibers, and average fiber length (in millimeters). The following values were obtained: fiber ratio (fiber ratio = number of fibers in the pathway * 100/number of fibers in the whole brain), fractional anisotropy, mean diffusion (MD), axial diffusion (AD) and radial diffusion (RD). In the study, the pain levels of the participants and tractography data of the pain pathways were collected.

### 2.4. Statistical analysis

The data were analyzed using the IBM SPSS Statistics 25 program (IBM Corp., Armonk), with a *P* value of <.05 deemed to be statistically significant. The data were summarized as mean ± standard deviation and percentage, and the chi-square test was employed to compare categorical data. Comparisons between 2 groups were made using the Student *t* test (*t* test for independent groups) when parametric conditions were met, and with the Mann–Whitney *U* test when they were not met.

## 3. Results

In our study, there were 38 individuals in both groups. No significant difference was found between the 2 groups in terms of age, weight, height, gender, and BMI values (*P* ˃ .05), (Table 1). Mean fiber length, MD, AD, and RD values related to the whole brain were found to be significantly higher in the DS group than in the control group (*P* < .05), (Table 2). Fiber number and fiber ratio of both right and left LM were found to be significantly lower in DS individuals (*P* < .05), (Table 2).

**Table 1:** Descriptive statistics of the participants.

	Study group	Control group	Sig. (*P*)
Gender F/M (%F/%M)	22/16 (%57.9/%42.1)	24/14 (%63.2/%36.8)	.358*
Age (yr)	58.31 ± 13.75	55.11 ± 5.44	.804**
Weight (kg)	73.89 ± 10.14	76.21 ± 10.77	.499
Height (m)	164.11 ± 6.79	160.47 ± 8.43	.153
BMI	28.38 ± 2.84	29.60 ± 3.56	.241
VAS	6 (2.50–9.50)	3.50 (3–7)	.275

BMI = body mass index, VAS = visual analog scale.

* Chi-square test.

** Mann–Whitney *U* test.

**Table 2:** Comparison of pain pathway tractography data and demographic information between groups.

	Degenerative (n = 38) (mean ± standard deviation)	Control (n = 38) (mean ± standard deviation)	Sig. (*P*)
All brain			
Fiber count	82,385.36 ± 1508.2	82,298.10 ± 964.59	.833
Mean fiber length	65.70 ± 2.7	63.51 ± 1.97	**.007**
Volume (mm^3^)	849,254.36 ± 63,681.07	853,220.52 ± 69,223.79	.855
Surface area (mm^2^)	468,132.57 ± 54,757.37	442,484.78 ± 35,985.59	.097
Fractional anisotropy (FA)	0.4 ± 0.01	0.4 ± 0.01	.294
Mean diffusivity (MD)	0.9 ± 0.03	0.87 ± 0.02	**.003** *
Axial diffusivity (AD)	1.31 ± 0.03	1.27 ± 0.02	**.001**
Radial diffusivity (RD)	0.70 ± 0.03	0.67 ± 0.02	**.004**
Left lemniscus medialis			
Fiber count	1506.36 ± 260.74	2727.31 ± 1443.21	**.001**
Mean fiber length	137.47 ± 8.52	133.63 ± 7.35	.146
Volume (mm^3^)	14,175.88 ± 2978.24	15,023.64 ± 3573.65	.274*
Surface area (mm^2^)	13,637.88 ± 2525.16	13,793.62 ± 2101.37	.837
Fractional anisotropy (FA)	0.49 ± 0.02	0.49 ± 0.02	.858
Mean diffusivity (MD)	0.85 ± 0.03	0.85 ± 0.03	.885
Axial diffusivity (AD)	1.34 ± 0.06	1.34 ± 0.05	.957
Radial diffusivity (RD)	0.6 ± 0.02	0.6 ± 0.03	.988
Fiber ratio	1.82 ± 0.3	3.30 ± 1.73	**.001** *
Volume ratio	1.68 ± 0.39	1.77 ± 0.46	.328*
Area ratio	2.95 ± 0.64	2.95 ± 0.64	.355
Right lemniscus medialis			
Fiber count	2094.78 ± 761.83	3332.31 ± 1502.19	**.011** *
Mean fiber length	133.34 ± 11.56	130.52 ± 8.64	.401
Volume (mm^3^)	16,373.98 ± 3708.04	15,364.58 ± 3513.08	.395
Surface area (mm^2^)	15,084.38 ± 2932.92	13,858.28 ± 2471.63	.112*
Fractional anisotropy (FA)	0.50 ± 0.02	0.50 ± 0.02	.902
Mean diffusivity (MD)	0.83 ± 0.02	0.84 ± 0.05	.759*
Axial diffusivity (AD)	1.33 ± 0.04	1.33 ± 0.04	.957
Radial diffusivity (RD)	0.58 ± 0.03	0.59 ± 0.05	.398
Fiber ratio	2.54 ± 0.92	4.04 ± 1.8	**.011** *
Volume ratio	1.91 ± 0.36	1.80 ± 0.4	.372
Area ratio	3.21 ± 0.41	3.13 ± 0.51	.618
Left formatio reticularis			
Fiber count	1323.26 ± 483.1	1144.94 ± 596.93	.175*
Mean fiber length	121.48 ± 7.96	112.71 ± 8.25	**.002**
Volume (mm^3^)	23,779.83 ± 4620.5	18,549.51 ± 6708.65	**.008**
Surface area (mm^2^)	25,250.28 ± 6249.87	20,415.87 ± 7755.86	**.041**
Fractional anisotropy (FA)	0.42 ± 0.03	0.43 ± 0.02	.725
Mean diffusivity (MD)	0.92 ± 0.06	0.90 ± 0.04	.140
Axial diffusivity (AD)	1.34 ± 0.06	1.32 ± 0.04	.339
Radial diffusivity (RD)	0.7 ± 0.06	0.69 ± 0.04	.332
Fiber ratio	1.62 ± 0.58	1.39 ± 0.73	.157*
Volume ratio	2.8 ± 0.54	2.17 ± 0.77	**.024** *
Area ratio	5.44 ± 1.42	4.59 ± 1.64	.099
Right formatio reticularis			
Fiber count	1262 ± 776.68	971.42 ± 362.94	.215*
Mean fiber length	118.44 ± 8.02	116.80 ± 8.89	.556
Volume (mm^3^)	17,571.55 ± 4767.41	16,761.48 ± 7287.90	.688
Surface area (mm^2^)	19,248.75 ± 4701.31	19,168.06 ± 8646.65	.972
Fractional anisotropy (FA)	0.44 ± 0.01	0.43 ± 0.02	.127
Mean diffusivity (MD)	0.95 ± 0.04	0.88 ± 0.03	**.001**
Axial diffusivity (AD)	1.32 ± 0.03	1.36 ± 0.07	**.015**
Radial diffusivity (RD)	0.73 ± 0.07	0.66 ± 0.04	**.001**
Fiber ratio	1.18 ± 0.45	1.53 ± 0.93	.215*
Volume ratio	1.97 ± 0.83	2.05 ± 0.49	.707
Area ratio	4.06 ± 1.65	4.37 ± 1.13	.497

Bold values indicate statistically significant difference observed in mean fiber length between degenerative and control groups (*P* = .007).

* Mann–Whitney *U* test.

The mean fiber length, fiber volume, fiber area, and fiber ratio values of the left RF were found to be significantly higher in the DS group (*P* < .05). The MD, AD, and RD values of the right RF were found to be significantly higher in the DS group (*P* < .05). At the same time, VAS values were significantly higher in the DS individuals compared to the control group (*P* < .05).

## 4. Discussion

In this study, the LM and RF pathways responsible for proprioceptive input and pain modulation in the central nervous system were analyzed in individuals with DS using tractography. The most remarkable finding is the significant differences in tractography metrics of both LM and RF pathways between individuals with DS and healthy controls.

Lower fractional anisotropy values indicate disruption of the myelin sheath surrounding axons, while elevated MD values reflect deterioration in white matter microstructure, potentially due to axonal or myelin damage. A decrease in AD is typically associated with axonal injury or reduced axonal caliber, whereas increased RD suggests demyelination or axonal loss. In addition, a reduced number of fibers may impair the functional integrity of these pathways.^[16–18]^

Spinal deformities in DS are known to disrupt postural balance and limit daily living activities.^[19]^ Key contributors to balance impairment include alterations in the center of gravity, muscle weakness, and asymmetric muscular activation.^[20]^ Spinal misalignment places a mechanical burden on postural control systems.^[21]^ For example, Haddas and Lieberman reported that individuals with adolescent DS exhibit greater postural sway in both sagittal and coronal planes during dynamic balance tasks compared to healthy controls.^[8]^ Furthermore, DS has been associated with altered gait patterns, reduced step length and joint range of motion, and asymmetry in trunk rotation and ground reaction forces.^[10,22,23]^ Given that spinal alignment and balance information are relayed to the brain via proprioceptive input,^[13]^ the observed decrease in LM fiber number and proportion in DS individuals suggests impaired transmission of proprioceptive signals to the cerebral cortex. This may contribute to deficits in neuromuscular control and postural stability. Within the complex interplay of suprapelvic and infrapelvic alignment parameters, diminished proprioceptive feedback may hinder the compensatory mechanisms needed to maintain balance.

Regarding pain, our findings are consistent with previous studies indicating that individuals with DS report more widespread and asymmetric pain.^[4]^ While pain in DS is most commonly localized in the lumbar region, it may also affect thoracolumbar or thoracic segments.^[23]^ In adolescent idiopathic scoliosis, pain is generally reported when the curvature exceeds 30°,^[23]^ whereas in DS, pain can occur even at lower degrees of curvature.^[24]^ Notably, in DS, the distribution of pain tends to be broader and less correlated with the structural severity or direction of spinal deformity.^[3,23,25]^

The RF is recognized for its critical role in modulating and suppressing nociceptive inputs.^[7,26]^ Due to its extensive connections with the cerebral cortex, diencephalon, basal ganglia, cerebellum, and spinal cord, the RF may contribute to the mismatch between the origin and perception of pain.^[7,27]^ In our study, individuals with DS showed increased fiber number and proportion in the right RF, and elevated fiber length, volume, area, MD, and RD values in the left RF, along with decreased AD. These changes suggest a structural adaptation of the RF in DS, potentially reflecting its enhanced role in chronic pain modulation. Alterations in RF axonal structure and myelination may underlie the increased frequency and wider distribution of pain in DS.

Although the present study focused specifically on degenerative scoliosis, spinal alignment abnormalities are not limited to coronal plane deformities. Similar central nervous system alterations may also be present in other spinal deformities such as kyphotic deformities or loss of normal spinal curvature. Future studies comparing different spinal deformity patterns may help determine whether the observed neural changes are specific to degenerative scoliosis or represent a general central adaptation to chronic spinal malalignment.

This study has several limitations. First, the relatively small sample size may limit the generalizability of the findings. Second, the cross-sectional design precludes causal inferences; therefore, it cannot be determined whether the observed tractography alterations are a cause or a consequence of degenerative scoliosis. Longitudinal studies evaluating neural microstructural changes before and during disease progression are required to clarify the temporal relationship between spinal deformity and central nervous system adaptations. In addition, the present study did not include pre and postoperative assessments. Prospective investigations examining tractography findings before and after deformity corrective surgery would provide valuable insight into whether the observed central sensory pathway alterations are reversible or represent long-term neuroplastic adaptations. Furthermore, this study focused exclusively on individuals with degenerative scoliosis. Therefore, the findings cannot be generalized to other scoliosis subtypes, such as congenital or idiopathic scoliosis, which differ in etiology, age of onset, and biomechanical characteristics. Comparative studies across different scoliosis subtypes may help determine whether the observed neural alterations are specific to degenerative mechanisms or reflect a broader central adaptation to spinal deformity. Finally, genetic evaluation was not performed. Although genetic factors have been implicated in certain scoliosis subtypes, particularly idiopathic scoliosis, potential genetic differences between the study and control groups cannot be excluded. Future multimodal studies integrating genetic profiling with advanced neuroimaging techniques may provide a more comprehensive understanding of central nervous system involvement in scoliosis.

This study highlights potential alterations in the LM and RF pathways related to proprioception and pain modulation in individuals with DS. The observed microstructural changes may help explain postural instability and altered pain perception in this population. These findings underscore the importance of further investigating proprioceptive pathway integrity and reticular system involvement in balance and pain management. Such insights could pave the way for novel rehabilitation strategies targeting central mechanisms in DS.

## Author contributions

**Conceptualization:** Sabri Batin.

**Data curation:** Yasin Göktürk.

**Investigation:** Yasin Göktürk.

**Methodology:** Yasin Göktürk, Şule Göktürk.

**Software:** Tahir Fatih Dikici, Ahmet Payas.

**Supervision:** Sabri Batin.

**Validation:** Tahir Fatih Dikici, Ahmet Payas.

**Visualization:** Tahir Fatih Dikici.

**Writing – original draft:** Şule Göktürk, Ahmet Payas.

**Writing – review & editing:** Şule Göktürk, Ahmet Payas.
